# Barriers and facilitators influencing the choice of a vegetarian menu in a university cafeteria

**DOI:** 10.1017/jns.2024.69

**Published:** 2024-11-18

**Authors:** Valeria A. Bertoni Maluf, Sidonie Fabbi, Carolina Cerqueira Azevedo, Isabelle Carrard

**Affiliations:** Department of Nutrition and Dietetics, Geneva School of Health Sciences, HES-SO University of Applied Sciences and Arts Western Switzerland, Carouge, Switzerland

**Keywords:** Barriers, Facilitators, Obstacles, Sustainable diets, Vegetarian menu, University

## Abstract

This cross-sectional study examined the barriers and facilitators that influence vegetarian menu choices in a university cafeteria in Geneva, Switzerland. As a first step, an online survey developed by the authors based on the Capability, Opportunity, and Motivation Behaviour (COM-B) model was e-mailed to all university students and staff. In the second step, focus groups (FG) were held to complete the survey responses and identify what needed to be changed to promote the choice of the vegetarian menu in the cafeteria. Data from 304 participants collected through the survey was analysed. The main mentioned barriers were lack of vegetarian options, tastelessness and insufficient satiation. The facilitators that emerged from the survey were the price of the vegetarian menu for students and health and environmental benefits. Thirteen people participated in four FG sessions, which were analysed using thematic analysis. Five themes were identified: spontaneous menu selection, predefined menu selection, influence of opportunity on menu selection, influence of environmental sensitivity on menu selection, and threat to identity in menu selection. The choice of a vegetarian menu in a university cafeteria was mainly influenced by the attractiveness and taste of the plate. Future strategies to reduce food-related greenhouse gas emissions should (a) ensure the quality and attractiveness of the vegetarian menu, especially to appeal to the more resistant, such as men and omnivores, and (b) inform consumers about the guarantee of balanced nutrient intake of the vegetarian menu offered in the cafeteria, and about health and environmental benefits.

## Introduction

The global surface temperature increased by approximately 1.1°C in the period 2011–2020 compared to 1850–1900^(1)^ and the year 2023 was the warmest year ever recorded on Earth.^(2)^ Global warming is undoubtedly a direct consequence of human activities, mainly greenhouse gas emissions (GHG emissions),^(3)^ and the current food system is responsible for approximately 30% of global emissions.^(4)^ In Switzerland, the percentage of food impact is 25%^(4)^ having red meat, dairy, and processed meat being the three food categories with the highest GHG emissions.^(5)^ Moreover, the National Nutrition Survey MenuCH 2014–2015 revealed that the Swiss adult population consumed an average of 111 g of meat per person per day, three times more than the recommended amount, with men and younger people being the biggest consumers.^(6)^ Several organizations have confirmed the link between diet, environmental impacts, and human health. The Food and Agriculture Organization (FAO) and World Health Organization (WHO) describe ‘sustainable healthy diets’ as diets that promote health and well-being, have low environmental pressure and impact, are accessible, affordable, safe, equitable, and culturally acceptable.^(7)^ The Intergovernmental Panel on Climate Change (IPCC) refers to ‘healthy and sustainable diets’ as a major opportunity to reduce GHG emissions from food systems and improve health outcomes.^(1)^ To ensure human health and environmental sustainability, the EAT-Lancet Commission has set intake targets for food groups (e.g. 14 g/d for beef, lamb, or pork; 29 g/d for chicken and other poultry).^(8)^ The Swiss Nutrition Society (SSN) also recommends a moderate intake of meat, chicken, and processed meat (2–3 times a week).^(9)^

In 2015, Switzerland signed the Paris Agreement, committing to halve its emissions by 2030 compared to 1990 to achieve net-zero emissions by 2050,^(10)^ and reducing meat consumption is one of the changes needed to meet these commitments. Even if meat consumption tends to decrease, with a decline of 5.9 per cent since 2014,^(11)^ it remains high and far from the recommendations.

Every day in Switzerland, around one million people eat in a collective catering establishment ^(12)^ (restaurant, bar, canteen, cafeteria), and according to the results of the Household Budget Survey 2020 around 4.6% of net income was spent on food and beverages between 2015 and 2019.^(13)^ Thus, a cafeteria can be a good place to promote a sustainable healthy diet. Nonetheless, food choices, particularly meat consumption behaviour, are complex and influenced by several factors, such as knowledge, values, emotions, social norms, perceived behavioural control, and the food environment.^(14)^ In addition, high prices or poor taste have been identified as barriers to making climate-friendly food choices, and the perception of barriers was specific according to gender and type of diet (vegetarians or meat and/or fish consumers).^(15)^ Therefore, consumer barriers and facilitators should be considered to effectively target and reduce meat consumption in cafeterias.

We aimed to identify the barriers and facilitators that influence the choice of vegetarian menu in a university cafeteria in Geneva, Switzerland. The choice of university was based on literature findings that a reduction in meat consumption is particularly necessary among 18- to 24-year-olds and men with a higher education.^(5)^ This study was the first step in a larger study that aimed to design an intervention in the form of an information campaign, using the Behaviour Change Wheel methodology as a framework.^(16)^ The Behaviour Change Wheel methodology has been designed to support the development of behaviour change intervention based on theory rather than on insight. The authors synthesized 19 frameworks into a new methodology that entails a ‘behaviour system’, the Capability, Opportunity, Motivation, and Behaviour (COM-B).^(17)^ The COM-B model describes the conditions necessary for a behaviour to occur in terms of psychological and physical capability, social and physical opportunity, and automatic and reflective motivation. It helps identify the relevant components to target when designing an intervention and to pair them with the adequate interventions and policies. The COM-B model, was used as a reference to guide us in examining the barriers and facilitators of the behaviour to be changed.

## Methods

### Study participants and location

This cross-sectional study took place at the University of Applied Sciences in Geneva, with more than 1’200 students and 413 staff members (teachers, administrative/technical staff) training students in agronomy, landscape architecture, and nature management.^(18)^ This university is responsible for training students in various fields (landscape, architecture, and engineering), which typically include more men than in other fields (e.g. health), and a majority of men with higher education.

The cafeteria of this university, run by a private company, is surrounded by a large number of restaurants and shops that offer an attractive range of food at discounted prices and must meet consumer expectations and contractual obligations with the university.

At the time of the study, the cafeteria was still undergoing some post-COVID-19 measures, such as reducing the menu choices offered, eliminating the salad buffet, and offering a discount price for students on a menu that was vegetarian by default and sold at CHF 5.- (approximately EUR 5.- or USD 5.-). The discount was more than half the normal price of the menu, which originally cost CHF 11.-. This measure was intended to prevent food insecurity among students. The cafeteria offered three main meal options: a meat or fish menu called the ‘Market menu’, a ‘Pizza of the Day’, and a vegetarian menu called the ‘Balance Menu’, which was certified as nutritionally balanced.

The study was conducted in two steps, with the primary outcome focused on identifying barriers and facilitators that influence the purchase of vegetarian menus in this population.

### Survey: 1st step

An online closed survey was conducted using LimeSurvey® and sent by e-mail to all students and staff members of the university, with the subject line of the e-mail invitation noting the survey was for students and staff who often visited the cafeteria (S1 of the Supplementary Material). The e-mail, which was distributed by the university administration via a mailing list, informed participants of the objectives of the study and the expected duration of the survey (approximately 10 minutes). Users were given direct access to the survey via a provided link, eliminating the need for registration. The survey was developed by the authors based on the context of the study, COM-B model^(17)^ and other questionnaires found in the literature.^(15,19,20)^ In brief, the survey included four sections and thirteen questions addressing the following: sociodemographic (e.g. age, gender, and function/role), types of diets (e.g. omnivore, vegetarian, flexitarian), recommendations for meat and fish consumption (awareness and opinion of the EAT-Lancet recommendations), and barriers and facilitators/benefits to choosing vegetarian menu in the cafeteria (including questions with a list of options about personal barriers and perceived facilitators/benefits to vegetarian menus, with the possibility to give ‘other’ answers not included in the list). The survey was initially piloted on five registered dieticians and a psychologist to check the clarity and wording of the questions, the time required to complete the survey, and to validate the established questions. Adjustments were then made. There were a total of 15 pages (one page per question) and 13 mandatory questions, with two optional questions, one to add suggestions and one to provide an e-mail address for those interested in participating in the focus groups (FG). All questions were asked of all participants, and the order of questions was the same for each participant. The survey was conducted in French, the official language of the canton, and data collection was conducted from April to May 2022. We used a convenience sample. Participation was voluntary and anonymous, and there was no compensation for students or staff to complete the survey. The reasons for non-participation could not be determined. At the end of the survey, participants who wanted to join the FG were asked to provide their e-mail addresses, and were contacted later. The survey announcement and the final model of the survey are presented in S1 and S2 of the Supplementary Material, respectively.

### Focus groups (FG): 2nd step

To complete the survey responses, identify what needed to change to promote the choice of the vegetarian menu in the cafeteria, and select the most appropriate intervention, the interview guide of FG was developed by the research team. The interview guide was based on the COM-B model, supplemented by the Theoretical Domains Framework (TDF),^(21–23)^ which allows for a deeper examination of the influences in the COM-B model. The domains of the TDF used to develop the questions were selected based on the survey responses: Memory, attention and decision process; Emotion; Beliefs about capabilities; and Environmental context and resources. In brief, the interview guide included a summary of the online survey results and then questions about whether participants felt represented in these results, whether they chose or intended to choose a vegetarian menu in the cafeteria, and the feeling of choosing the vegetarian menu in the cafeteria. As the ultimate goal of the main study was to design an intervention based on an information campaign, the last part of the interview was dedicated to the question of which messages would be suitable for cafeteria customers and which media would be better. The same script was used for all the sessions. The final model of the interview guide is presented in S3 of the Supplementary Material.

To include participants in the FG, we selected non-vegetarians who were available (*n* = 13) from among those who expressed interest in the questionnaire (*n* = 35). The rationale for selecting only non-vegetarians (omnivores and flexitarians) was to gain insight into the barriers and motivators that influence their menu choices. Vegetarians already tend to choose the vegetarian menu based on their dietary preferences or convictions, which would not provide us with the necessary information on why non-vegetarians might resist or accept the vegetarian options. Understanding the perspective of non-vegetarians is crucial, as they represent the majority of cafeteria customers. This approach allows us to identify specific factors that can be targeted in interventions to promote vegetarian choices. The FG sessions took place in a university classroom on four different days between May and June 2022, and each participant chose the most convenient date according to their availability. Three of the FG were led by two dieticians and one by a dietician and psychologist. VABM, a dietician with over 10 years of experience and a research assistant with a master’s degree in health sciences, was responsible for facilitating all discussions. She was assisted by a second person, either a research assistant with a BSc in Nutrition and dietetics, or in one case, a professor with a PhD in psychology (IC), who took notes and recorded participants’ nonverbal expressions. The sessions were held during lunch breaks and lasted for an hour. Participants were seated in a circle to encourage the exchange of ideas and were provided with a lunch box (they could choose their sandwich in advance, either vegetarian or not) for consumption during the discussion. The participants were informed in advance that each session was audio-recorded. Participants were offered a voucher worth CHF 20 (∼ EUR 20/USD) in the cafeteria as compensation for their participation.

With regard to potential sources of bias, the researchers conducting the study were not affiliated with either the university or the cafeteria, and no other interventions were introduced in the cafeteria during the study period, minimizing potential external influences on the research results.

The study adhered to the guidelines outlined in the Declaration of Helsinki. Verbal informed consent was obtained from all the participants in the FG. The cantonal ethics committee (Geneva CCER) confirmed that this study did not fall under the Swiss Federal Law on Human Research and did not request review (Req-2021-01395). Although a protocol was developed and reviewed for grant submission, it has not been published.

### Data analyses

Frequencies and percentages, means and standard deviations were calculated for the data collected in the survey from the selected participants. Comparisons between males and females, and between students and staff members were performed using Fisher’s exact tests. Comparisons between the various diet types (omnivores, flexitarians, and vegetarians grouped with vegans) were calculated using Pearsons’s chi-squares. Seven (2%) participants declared to belong to another gender category and were not included in the gender analyses. Research/teaching staff and administrative/technical staff were grouped and compared to student responses. Differences were considered significant at the 5% level. Quantitative data were analysed using SPSS® for Windows, version 26.0, 2019 (IBM®).

FG recordings were anonymously transcribed verbatim by VABM and verified by IC. Transcripts were then analysed using thematic analysis, as proposed by Braun and Clarke,^(24)^ using NVivo software (version 1.6.1, QSR International). Reflexive thematic analysis was used, which means that the researchers worked to generate the themes and acknowledged their subjectivity.^(25)^ This approach was chosen because the ultimate goal was to design an intervention based on the Behaviour Change Wheel methodology, and thematic analysis allows for theoretical flexibility.^(26)^ IC and VABM both coded the data, first in a very general way, and then refined the coding with barriers and facilitators in mind. The data corpus was coded inductively according to the topics discussed in the FG and deductively using the COM-B model as a reference, i.e. barriers and facilitators were searched for, keeping in mind a possible classification into Capability, Opportunity of Motivation. IC is a psychologist and VABM is a dietitian; both define themselves as flexitarians. This was their first foray into research on the environmental impact of food, and they were naive about the ideological conflicts underlying the issue. After the coding process, the researchers collaborated to generate themes that reflected how the participants chose their menu in the cafeteria, including barriers and facilitators.

## Results

### Survey completion

A total of 376 participants opened the questionnaire, and 22 of them closed it without answering any question. Of the remaining 354 participants, 10 stopped after the sociodemographic questions, 12 more after the diet type questions, and 28 more after the recommendation questions. Twenty-nine participants then stopped somewhere in the barriers and facilitators questions, but because they had answered at least one of these four questions and because it was our topic of interest, we included them in our analyses. We therefore decided to analyse the data from these 304 participants. In the end, 271 completed the 15 pages to the end (completion rate of 72%), after four abandonments at page 14.

### Survey: participants’ characteristics

Of the 304 participants whose data were considered, the majority were male (*n* = 181, 60%). Sixty-four per cent of respondents were students (*n* = 195), 22% were research and teaching staff (*n* = 66), and 14% were administrative and technical staff (*n* = 43). The average age of the participants was 30.6 SD 13.1.

Most of the participants said they were omnivores (*n* = 176, 58%), 33% flexitarians (*n* = 101), 8% vegetarians (*n* = 25), and less than 1% vegans (*n* = 2, 0.7%). More than a third of the respondents said that there were no barriers (41%, *n* = 124) to choosing the vegetarian menu in the cafeteria, and around 80% of participants had the intention (*n* = 246, 82%) and value (*n* = 249, 82%) (yes and rather yes) to adopt a sustainable diet. A substantial number declared already had reduced (*n* = 128, 42%) or stopped (*n* = 26, 9%) eating meat and fish. On the other hand, 20% of respondents (*n* = 60) did not intend to reduce their meat consumption because they enjoyed eating meat, they did other things for the environment, or it was too restrictive.

Regarding the EAT-Lancet recommendations, more than half of the participants were unfamiliar with them (*n* = 193 votes, 64%), and only a small number of people said they were aware of and respected the recommendations (*n* = 22 votes, 7%).

Moreover, in the free comments, a considerable number of participants (*n* = 30, 10% of the respondents) reported strongly opposed reactions to the topic. One group of people, already vegetarians, felt that the cafeteria should stop serving animal flesh and become ‘meat-free’. Another group of people opposed the promotion of the vegetarian menu as well as the study.

### Survey: barriers and facilitators to choosing the vegetarian menu in the cafeteria

Tables 1 and 2 show participants’ reported barriers and facilitators to choosing the vegetarian menu in the cafeteria, mentioned by more than 5% of the participants, by gender. Table 3 shows the reported barriers and facilitators according to the type of diet self-reported by the participants.

**Table 1.** Barriers to choosing the vegetarian menu in the cafeteria, by gender (total *n* = 297, *n* = 116 women and *n* = 181 men)

Barriers/Obstacles^a^	All^b^ *n* = 297 (100%)	Women *n* = 116 (100%)	Men *n* = 181 (100%)	P value^c^
No barriers	121 (40.7)	62 (53.4)	59 (32.6)	<0.001^*^
Too few vegetarian options	124 (41.8)	51 (44.0)	73 (40.3)	0.549
Tastes less good	90 (30.3)	23 (19.8)	67 (37.0)	0.002^*^
Insufficiently satiating	71 (23.9)	16 (13.8)	55 (30.4)	0.001^*^
Enjoy eating meat or fish in the cafeteria	58 (19.5)	15 (12.9)	43 (23.8)	0.024^*^
High price in relation to energy provided	46 (15.5)	11 (9.5)	35 (19.3)	0.022^*^
Worry about deficiencies (iron, vit. B12)	42 (14.1)	11 (9.5)	31 (17.1)	0.087
Worry about not getting enough protein	42 (14.1)	10 (8.6)	32 (17.1)	0.040^*^
Take advantage of eating meat or fish in the cafeteria because it is affordable	40 (13.5)	18 (15.5)	22 (12.2)	0.486
Don’t have the habit	36 (12.1)	9 (7.8)	27 (14.9)	0.071
Tired of being told what to do and what not to do	34 (11.4)	7 (6.0)	27 (14.9)	0.024^*^
Whatever your diet, the planet will not be saved	34 (11.4)	9 (7.8)	25 (13.8)	0.136
Think that human beings were meant to eat meat and fish, to evolve	33 (11.1)	7 (6.0)	26 (14.4)	0.036^*^
Lack of willpower to stop meat/fish consumption	28 (9.4)	5 (4.3)	23 (12.7)	0.015^*^
Lack of knowledge about the substitution of meat/fish at the buffet	23 (7.7)	7 (6.0)	16 (8.8)	0.505
Fear of losing muscle	20 (6.7)	2 (1.7)	18 (9.9)	0.007^*^
Having so many things on your mind that by the time you get to the cafeteria, you forget your resolutions to eat more sustainably	19 (6.4)	6 (5.2)	13 (7.2)	0.629
Other answers	< 5%			

a More than one answer was possible.

b 7 (2%) declared to be in another gender category.

c Fisher’s exact test.

* P < 0.05.

**Table 2.** Facilitators to choosing the vegetarian menu in the cafeteria, by gender (total *n* = 297, *n* = 116 women and *n* = 181 men)

Facilitators^a^	All^b^ *n* = 297 (100%)	Women *n* = 116 (100%)	Men *n* = 181 (100%)	P value^c^
Eat more fruit and vegetables with the vegetarian menu	167 (56.2)	70 (60.3)	97 (53.6)	0.281
Less environmental impact	151 (50.8)	63 (54.3)	88 (48.6)	0.344
As the vegetarian menu is often priced at CHF 5, I take it more regularly	126 (42.4)	53 (45.7)	73 (40.3)	0.400
Allows you to spend less money	114 (38.4)	41 (35.3)	73 (40.3)	0.396
Contributes to animal rights and animal welfare	86 (29.0)	42 (36.2)	44 (24.3)	0.036^*^
Helps to improve health through the reduction of negative products in meat (chemicals, steroids and antibiotics)	85 (28.6)	30 (25.9)	55 (30.4)	0.432
The satisfaction of doing something for the planet (the environment or the welfare of animals)	80 (26.9)	36 (31.0)	44 (24.3)	0.228
The quality of the fat is better	72 (24.2)	26 (22.4)	46 (25.4)	0.582
Helps to prevent certain types of disease	65 (21.9)	19 (16.4)	46 (25.4)	0.084
Improvement in the efficiency of food production and hence food security	55 (18.5)	21 (18.1)	34 (18.8)	1.000
Nothing makes that choice easier	52 (17.5)	12 (10.3)	40 (22.1)	0.012^*^
Allows you to control your weight	44 (14.8)	17 (14.7)	27 (14.9)	1.000
Helps you to stay fit and full of energy	38 (12.8)	16 (13.8)	22 (12.2)	0.723
No profit	27 (9.1)	6 (5.2)	21 (11.6)	0.065
Allows you to have a tastier diet	27 (9.1)	16 (13.8)	11 (6.1)	0.037^*^
Choose a vegetarian option as the wait in the cafeteria is shorter	20 (6.7)	11 (9.5)	9 (5.0)	0.156
Other answers	< 5%			

CHF, Swiss Franc.

a More than one answer was possible.

b 7 (2%) declared to be in another gender category.

c Fisher’s exact test.

* P < 0.05.

**Table 3.** Significant barriers and facilitators to choosing the vegetarian menu in the cafeteria by diet type (*n* = 304)

Barriers — Facilitators^a^	Omnivore (*n* = 176, 100%)	Flexitarian (*n* = 101, 100%)	Vegetarian vegan (*n* = 27, 100%)	P value^b^
Barriers				
No barriers (*n* = 124, 40.8%)	51 (29.0%)	56 (55.4%)	17 (63.0%)	<0.001^*^
Tastes less good (*n* = 92, 30.3%)	68 (38.6%)	22 (21.8%)	2 (7.4%)	<0.001^*^
Insufficiently satiating (*n* = 72, 23.7%)	51 (29.0%)	19 (18.8%)	2 (7.4%)	0.018 ^*^
Enjoy eating meat or fish in the cafeteria (*n* = 59, 19.4%)	52 (29.5%)	7 (6.9%)	0 (0.0%)	<0.001^*^
Worry about deficiencies (iron, vit. B12) (*n* = 44, 14.5%)	34 (19.3%)	9 (8.9%)	1 (3.7%)	0.015^*^
Worry about not getting enough protein (*n* = 43, 14.1%)	34 (19.3%)	7 (6.9%)	2 (7.4%)	0.010^*^
Don’t have the habit (*n* = 36, 11.8%)	33 (18.8%)	3 (3.0%)	0 (0.0%)	<0.001^*^
Tired of being told what to do and what not to do (*n* = 35, 11.5%)	34 (19.3%)	1 (1.0%)	0 (0.0%)	<0.001^*^
Think that human beings were meant to eat meat and fish, to evolve (*n* = 34, 11.2%)	30 (17.0%)	4 (4.0%)	0 (0.0%)	<0.001^*^
Lack of willpower to stop meat/fish consumption (*n* = 28, 9.2%)	26 (14.8%)	2 (2.0%)	0 (0.0%)	<0.001^*^
Facilitators				
Eat more fruit and vegetables with the vegetarian menu (*n* = 170, 55.9%)	80 (45.5%)	74 (73.3%)	16 (59.3%)	<0.001^*^
Less environmental impact (*n* = 154, 50.7%)	61 (34.7%)	70 (69.3%)	23 (85.2%)	<0.001^*^
As the vegetarian menu is often priced at CHF 5, I take it more regularly (*n* = 127, 41.8%)	63 (35.8%)	51 (50.5%)	13 (48.1%)	0.045^*^
Helps to improve health through the reduction of negative products in meat (chemicals, steroids and antibiotics) (*n* = 88, 28.9%)	36 (20.5%)	42 (41.6%)	10 (37.0%)	<0.001^*^
Contributes to animal rights and animal welfare (*n* = 87, 28.6%)	23 (13.1%)	44 (43.6%)	20 (74.1%)	<0.001^*^
The satisfaction of doing something for the planet (the environment or the welfare of animals) (*n* = 80, 26.3%)	21 (11.9%)	43 (42.6%)	16 (59.3%)	<0.001^*^
The quality of the fat is better (*n* = 73, 24%)	35 (19.9%)	27 (26.7%)	11 (40.7%)	0.045^*^
Helps to prevent certain types of disease (*n* = 65, 21.4%)	28 (15.9%)	29 (28.7%)	8 (29.6%)	0.024^*^
Improvement in the efficiency of food production and hence food security (*n* = 55, 18.1%)	11 (6.3%)	30 (29.7%)	14 (51.9%)	<0.001^*^
Nothing makes that choice easier (*n* = 54, 17.8%)	48 (27.3%)	4 (4.0%)	2 (7.4%)	<0.001^*^
Helps you to stay fit and full of energy (*n* = 40, 13.2%)	13 (7.4%)	20 (19.8%)	7 (25.9%)	0.002^*^
No profit (*n* = 28, 9.2%)	27 (15.3%)	1 (1.0%)	0 (0.0%)	<0.001^*^
Allows you to have a tastier diet (*n* = 27, 8.9%)	8 (4.5%)	11 (10.9%)	8 (29.6%)	<0.001^*^

a More than one answer was possible. To be included in Table 3, barriers and facilitators had to be cited by more than 5% of the participants.

b Pearson’s chi-square.

* P < 0.05.

Regarding barriers, among all participants (*n* = 304), ‘too few vegetarian options’ (*n* = 129, 42%), ‘tastes less good’ (*n* = 92, 30%), and ‘insufficiently satiating’ (*n* = 72, 24%) were the three most cited arguments, with more than 20% of votes. In contrast, ‘eat more fruit and vegetables’ (*n* = 170, 56%), ‘less environmental impact’ (*n* = 154, 51%), and ‘price’ (vegetarian menu at CHF 5.-: *n* = 127, 42%; and spending less money: *n* = 117, 39%) were cited as the main reasons for choosing a vegetarian menu.

Some other facilitators received more than 20% of the votes. They were related to health: ‘improving health by reducing negative products in meat’ (*n* = 88 (29%), and ‘better quality of fat’ (*n* = 73, 24%), and the environment/ethics: ‘contribution to animal rights and animal welfare’ (*n* = 87, 29%), and ‘satisfaction of doing something for the planet’ (*n* = 80, 26%).

Significantly more men than women reported that barriers to choosing a vegetarian menu were related to the following factors: less taste (37% vs. 20%; P = 0.002), insufficient feeling of satiety (30% vs. 14%; P = 0.001), price too high related to the energy provided (19% vs. 10%; P = 0.022), insufficient protein intake (17% vs. 9%; P = 0.040), and fear of losing muscle mass (10% vs. 2%; P = 0.007). The difference between men and women was also significant in terms of the enjoyment of eating meat/fish in the cafeteria (24% vs. 13%; P = 0.024), the belief that people should eat meat/fish (14% vs. 6%; P = 0.036), the unwillingness to stop eating meat/fish (13% vs. 4%; P = 0.015), and being tired of being told what they should do (15% vs. 6%; P = 0.024). It was mainly women who believed that there were no barriers to choosing a vegetarian menu in the cafeteria (53% vs. 33%, P < 0.001).

Only three facilitators for choosing the vegetarian menu in the cafeteria differed significantly between the men and women. For more women than men, the choice of the vegetarian menu was facilitated as it contributed to the respect for animal rights (36% vs. 24%; P = 0.036) and to the consumption of a tastier diet (14% vs. 6%; P = 0.037). Twenty-two per cent of men stated that nothing made it easier to choose a vegetarian menu in the cafeteria (vs. 10% of women; P = 0.012).

A comparison of the answers of the students (*n* = 195, 64%) with those of the employees (*n* = 109, 36%) showed only a few differences (Supplementary Table 1). More students than employees stated that they took advantage of spending less money (*n* = 91, 47% students vs. *n* = 26, 24% employees; P < 0.001), that they chose the vegetarian menu more often because the price was CHF 5.- (*n* = 110, 56% students vs. *n* = 17, 16% employees; P < 0.001) and that they took advantage of eating meat or fish in the cafeteria because it was affordable (*n* = 35, 20% students vs. *n* = 6, 6% employees; P = 0.003). In addition, more students were concerned about losing muscle (*n* = 19, 10% students vs *n* = 1, 1% employee; P = 0.003) and thought that there were not enough proteins in the vegetarian menu (*n* = 34, 17% vs *n* = 9, 8%, P = 0.038); however, more employees thought that the vegetarian menu helped prevent certain diseases (*n* = 31 employees, 28% vs. *n* = 34 students, 17%; P = 0.029). More employees mentioned as a facilitator that the wait in the cafeteria was shorter for the vegetarian menu (*n* = 12 employees, 11% vs. *n* = 8 students, 4%; P = 0.028), and that nothing made that choice easier (*n* = 29 employees, 27% vs. *n* = 25 students, 13%; P = 0.004).

When participants were compared according to their diet type (omnivores, flexitarians, vegetarians/vegans), it was found that most barriers were reported by omnivores, whereas most facilitators were reported by flexitarians and vegetarians/vegans (Table 3). The lack of taste (*n* = 68 omnivores, 39% vs *n* = 22 flexitarians, 22% vs *n* = 2 vegetarians/vegans, 7%; P < 0.001) and of satiation (*n* = 51 omnivores, 29% vs *n* = 19 flexitarians, 19% vs *n* = 2 vegetarians/vegans, 7%; P = 0.018) of the vegetarian menu, the worry about not getting enough micro- (*n* = 34 omnivores, 19% vs *n* = 9 flexitarians, 9% vs *n* = 1 vegetarians/vegans, 4%; P = 0.015) and macronutrients (*n* = 34 omnivores, 19% vs *n* = 7 flexitarians, 7% vs *n* = 2 vegetarians/vegans, 7%; P = 0.010), the lack of habit (*n* = 33 omnivores, 19% vs *n* = 3 flexitarians, 3% vs *n* = 0 vegetarians/vegans, 0.0%; P < 0.001) and/or willpower (*n* = 26 omnivores, 15% vs *n* = 2 flexitarians, 2% vs *n* = 0 vegetarians/vegans, 0.0%; P < 0.001), the tiredness of being told what to do (*n* = 34 omnivores, 19% vs *n* = 1 flexitarians, 1% vs *n* = 0 vegetarians/vegans, 0.0%; P < 0.001) and the belief that meat is necessary for human beings (*n* = 30 omnivores, 17% vs *n* = 4 flexitarians, 4% vs *n* = 0 vegetarians/vegans, 0.0%; P < 0.001) were barriers most often cited by omnivorous participants. The impact of the vegetarian menu on health, with the consumption of more fruits and vegetables (*n* = 80 omnivores, 46% vs *n* = 74 flexitarians, 73% vs *n* = 16 vegetarians/vegans, 59%; P < 0.001) and the environment (*n* = 61 omnivores, 35% vs *n* = 70 flexitarians, 70% vs *n* = 23 vegetarians/vegans, 85%; P < 0.001), as well as animal welfare (*n* = 23 omnivores, 13% vs *n* = 44 flexitarians, 44% vs *n* = 20 vegetarians/vegans, 74%; P < 0.001), were cited as facilitators, particularly by flexitarian and vegetarian/vegan participants.

### Focus groups: participants’ characteristics

A total of 13 people aged between 23 and 64 participated in the FG. The first FG consisted of one male student, although two other participants were scheduled, they did not attend. The second FG had four participants: one male student, two female students and one male teacher. The third FG included two male teachers and two assistants (one male and one female). The fourth FG consisted of two male research team members and two administrative/technical staff (one male and one female). Participants’ characteristics are presented in Table 4, with age presented in age ranges to prevent participant identification. Over 50% of participants (*n* = 8) were 40 years old or younger, and only two participants were in the 20–25 age group.

**Table 4.** Participants’ characteristics in focus groups (*n* = 13)

Participants — FG session	Gender	Age categories (years)	Position
P1 — 1^st^ FG	Male	20–25	Student
P2 — 2^nd^ FG	Male	26–30	Student
P3 — 2 ^nd^ FG	Female	20–25	Student
P4 — 2^nd^ FG	Male	41–45	Teacher
P5 — 2^nd^ FG	Female	26–30	Student
P6 — 3^rd^ FG	Male	56–60	Teacher
P7 — 3^rd^ FG	Male	26–30	Assistant
P8 — 3^rd^ FG	Female	26–30	Assistant
P9 — 3^rd^ FG	Male	31–35	Teacher
P10 — 4^th^ FG	Male	61–65	Research team
P11 — 4^th^ FG	Female	46–50	Administrative/technical staff
P12 — 4^th^ FG	Male	56–60	Administrative/technical staff
P13 — 4^th^ FG	Male	36–40	Research team

### Focus groups: thematic analysis of the FG

Five themes were identified using thematic analysis, which explained how the participants made menu choices in the cafeteria (Table 5). Each theme included barriers and facilitators of vegetarian menu choice.

**Table 5.** Generated themes in focus groups with examples

Themes	Examples of verbatims
Theme 1: Spontaneous menu selection	‘*It’s a bit of a global thing, I don’t necessarily think, it’s more about feelings*’ (P2).
Theme 2: Predefined menu selection	‘*At the moment I am also in the process of reducing my meat consumption and in fact in general I eat less than before, but not so long ago. A year ago I ate meat at every meal and then no, it’s a habit that becomes daily and I don’t really ask myself the question*’ (P4).
Theme 3: Influence of opportunity on menu selection	‘*Because there is the menu at 5 francs for students, 5 francs, and generally it’s vegetarian. So the students will eat vegetarian. Again, we are not attracting them by quality, but by price*’ (P12).
Theme 4: Influence of environmental sensitivity on menu selection	‘*When I choose a menu, it’s not so much for the environment as it is for me. Often I don’t choose meat because we have more than enough and I don’t need it for my health. But it’s more for me than for the environment* ’ (P4).
Theme 5: Threat to identity in the menu selection	‘*Again, just because you eat a vegetarian meal doesn’t mean you’re a vegetarian*’ (P6).

### Focus groups: theme 1 ‘spontaneous menu selection’

A spontaneous choice of the most attractive dish was the main reason for explaining the choice of one’s menu in the cafeteria. That spontaneous selection was a barrier to choosing the vegetarian menu, which was seen as less fancy and not in line with participants’ eating habits. Attention to the attractiveness of the vegetarian menu could make its choice more common.

Spontaneous menu selection was the main theme explaining how the participants chose their menu in the cafeteria: ‘*It’s a bit of a global thing, I don’t necessarily think, it’s more about feelings*’ (P2). Several factors were considered: the presentation of the menu was appetizing: *‘…the appearance of the plate in itself, even if in the end it’s the same plate with the same nutrients and the same food, depending on how it’s presented I find it’s just a kind of attractiveness trigger in fact*’. (P9); the menu had to be attractive, and participants had expectations about the pleasure they would feel: ‘*The priority is really to make me happy in fact. In addition, if we have a little stressful day, etc., this is the time when we calm down, we take the time to eat something good*’ (P11). It was mainly a barrier to choosing the vegetarian menu, which was poorly presented and less visually appealing than the non-vegetarian menu: ‘*I find that here there is a difference in the appeal of the non-vegetarian menu and the vegetarian menu just in the presentation, in the apparent quality of the dish*’ (P7).

The habit of choosing a menu type also contributed to the spontaneity of the choice and was also a barrier to the choice of the vegetarian menu: *‘…we got used to it, we actually have a plate, what is it: it’s steak, fries, vegetables. It can be something other than fries but there is a meat, a starch, a vegetable. And that’s the plate, let’s say typical. If we are given a vegetarian dish and then we only have the starch and the vegetable, we will say that something has been taken away from us. We are missing something*’ (P10).

The vegetarian menu in the cafeteria was judged to be unhealthy and unappetizing, and according to the participants, offering a vegetarian menu that is ‘real vegetarian food’ that looks appealing and good would help the cafeteria customers try it and get into the habit of choosing it: ‘*Because if he [the cook] does things well, we’ll all go vegetarian, in quotes, except that if he does bad things, well, that’s not going to encourage us to go vegetarian*’ (P12). Good vegetarian food-tasting stands could also inspire cafeteria customers to try new foods and change their habits: ‘*So I think you really have to inspire people with products that might be new. A few years ago we had vegetables from that era, things that I had never seen myself, that were super good, that we had tasted*’ (P11).

### Focus groups: theme 2 ‘predefined menu selection’

A facilitator of the vegetarian menu choice was that the choice was made in advance for health or environmental reasons. However, some participants lacked knowledge or had misrepresentations about the vegetarian menu, which did not encourage them to make this choice in their lives. Visual information may help them choose vegetarian menus. However, in the end, the information did not seem to be superior to the attractiveness of the menu in encouraging choices.

Predefined menu selection was the second theme, explaining how the participants chose their menu in the cafeteria. Some of the participants were trying to reduce their meat consumption for environmental or health reasons, and having made this decision in advance allowed it to become a habit: ‘*At the moment I am also in the process of reducing my meat consumption and in fact in general I eat less than before, but not so long ago. A year ago I ate meat at every meal and then no, it’s a habit that becomes daily and I don’t really ask myself the question*’ (P4). This decision, made in advance, facilitated the choice of vegetarian menu. However, the quality of the vegetarian menu could be discouraging even for those who had the intention to choose it: ‘*I eat quite regularly in the cafeteria with people from my lab and we have developed a bit of a joke around the vegetarian plate, which we often take because we all try to be careful, but which we sometimes avoid because we call it the “pig stuffing,” because it’s unfortunately a little too often, it doesn’t make you want to eat it at all, it’s recipes that are repeated a lot and which are unimpressive, unattractive…*” (P7).

Those who mainly chose their menu spontaneously were asked what kind of information they needed to make a more informed choice and arrive at a predefined menu selection. They suggested that nutritional information (calories, macronutrients) or information about the origin of the food would help their choice: ‘*It might be interesting to say that this plate is equivalent to a steak in terms of protein, but with fewer calories than a gratin*’ (P13) — ‘ *To have information and to be able to have a self-criticism or to say to oneself: oh, I eat a lot of pasta, shouldn’t I change my diet, it’s true that I don’t eat a lot of vegetables, what do vegetables bring me, yes, but vegetables are not good or but, I don’t know, and really to have information for young people, how to eat well?’* (P8). The information had to be simple and visual, such as the Nutri-score^1^, but for sustainability: ‘*So if there is a Nutri-score or something equivalent and well I find that in fact it has a big value. Eating something that is red, F, it’s like we start to say to ourselves: well, maybe not in fact, rather take thing A there, it will be better, or B’*.

Participants also mentioned the need for information that reflected misrepresentations, for example, the presence of genetically modified organisms (GMOs) in food, whereas in Switzerland, GMOs are so regulated that they are absent from the food sold: ‘*If I have the choice between, I mean, corn with GMO or without GMO, I choose without GMO’* (P11), or that the vegetarian menu sold in the cafeteria was not balanced and was not filling enough, whereas all vegetarian menus in the cafeteria followed a balanced diet charter: ‘*In the obstacles* [to choose the vegetarian menu], *the lack of a little bit of variety, and that there can even sometimes be the absence of proteins. Sometimes it’s just a little bit like a meat dish but without the meat*’ (P4).

They also asked for information that was already there and that they had not noticed, which shows that no information can reach its goal if the motivation is not internal: ‘P2: *There is really no indication of* [the origin]*…* P4: *yes, yes there is* P2: *Oh yeah?* P4: *yes, by chance, I saw it yesterday, because now there’s a screen at the entrance, they put a screen, and it said that there was GRTA* ^*2*^*…*’. In the end, spontaneity prevailed: ‘*I eat with my eyes first, if this vegetarian plate, it can be GRTA, proximity, sustainable, organic, if it doesn’t make me want it, it doesn’t make me want it. So it’s not a matter of communication*’ (P12).

### Focus groups: theme 3 ‘influence of opportunity on menu selection’

Among the opportunities that influenced the choice of vegetarian menu, the reduced price for students was one of the main facilitators. Another facilitator was the possibility of composing one’s own plate. Among the barriers, the lack of attractiveness of the vegetarian menu was again mentioned, and the lack of a salad buffet.

The reduced price for students was the main facilitator mentioned as motivating the choice of the vegetarian menu: ‘*The price is attractive, yes. Having the vegetarian menu at 5 francs. It’s…it motivates you to take it. Sometimes it allows you to have a dessert on the side with it*’ (P1). However, this reduction in price was also seen as a barrier by those who were not benefiting from it, because it reduced the quality of the vegetarian menu: ‘*Because there is the menu at 5 francs for students, 5 francs, and generally it’s vegetarian. So the students will eat vegetarian. Again, we are not attracting them by quality, but by price*’ (P12).

Among the other opportunities that facilitated the vegetarian menu, the participants mentioned the possibility of composing their plates with starch and vegetables. A salad buffet was also seen as a facilitator but had been removed due to COVID-19 restrictions: ‘*But at that time you could take salads by weight, and that’s great, and especially when it’s hot, it’s really nice to be able to say to yourself: ok, well, the vegetarian menu doesn’t tempt me more than that, well, I’ll have a salad*’ (P4).

The lack of attractiveness of the vegetarian menu was an obstacle to choosing it, even for those who had the intention to do so: ‘*I would like very much that I could not eat meat at lunch but unfortunately sometimes I choose the meat option, really reluctantly, because it is less bad*’ (P13). Participants regretted that the vegetarian menu in the cafeteria did not reflect the vegetarian cuisine eaten in restaurants, which would be much more appealing: ‘*In fact, what I find unfortunate is that the vegetarian menus are not at all representative of vegetarian cuisine to me. In fact, they are the image of people who can’t stop eating meat, of what they imagine to be a vegetarian dish*’ (P5).

### Focus groups: theme 4 ‘influence of environmental sensitivity on menu selection’

The environment was a sensitive issue that could be used as a facilitator to encourage the choice of vegetarian menus. However, despite concerns about this issue, at the time of the study, it was more of a barrier than a facilitator in choosing the vegetarian menu. Participants felt that sustainability was a complex issue, that it was used as a sales argument, and that they did not trust these allegations. Some participants also mentioned that their menu choices would not change anything about environmental issues. The origin of food was considered more important than eating meat. Participants also mentioned other initiatives they were taking to help the environment.

In general, the participants chose their menu according to their preference rather than because they were thinking about the environment, even if they chose the vegetarian menu: ‘*When I choose a menu, it’s not so much for the environment as it is for me. Often I don’t choose meat because we have more than enough and I don’t need it for my health. But it’s more for me than for the environment*’ (P4).

The participants were suspicious of the claims of sustainability of the vegetarian menu and considered the issue to be particularly complex: ‘*We talked about sustainable development, which of course plays a role in the environment, but at the same time we are focusing on the vegetarian menu. It bothers me because there is no agriculture without livestock, okay, so a strictly vegetarian diet, that’s my point of view, in any case from an agronomic point of view, it doesn’t make sense*’ (P6). Participants questioned whether the vegetarian menu was really good for the environment and not just an attempt at greenwashing: ‘*It’s really marketing to say sustainable menu and then to bring stuff from I don’t know where*’ (P3). The origin of food was seen as more important than eating meat: ‘*Well, I find that for me there is also the origin of food that we don’t necessarily have any information about and, I don’t know if there is a quinoa plate, but the quinoa comes from a long way away, while maybe the chicken comes from Geneva, I will say to myself ah bah, maybe I’ll have the chicken*’ (P8). Measures to reduce the meat consumption of the population were even seen as the wrong fight: ‘*I think there is a confusion in the term, when we think of the environment it is clearly not just meat. I also think that we have no way of knowing where the vegetables come from or anything like that. It’s not at all… for me, if I eat local, it’s out of concern for the environment. I don’t think so, it’s not just meat by far*’ (P5).

Some participants were less concerned about environmental issues, or were resigned and thought that their choice of menu at lunch would not change anything to the problematic: ‘P11: *Yes, but the information to say in this menu here we saved I don’t know, 3 litres of fuel…* P12: *After we are on vacation and we take the plane, what does it change*?’. Other participants mentioned other initiatives that they took out of concern for the environment, such as avoiding fish or seafood produced under unknown conditions: ‘*For example, on the contrary, I know that if there are shrimps, I will never take that menu, I have no idea where it comes from and then it’s a disaster, it’s really overfishing. So I try, although I really love fish, but I say to myself: well, if I’m not by the sea, I try to avoid it* ’ (P3).

### Focus groups: theme 5 ‘threat to identity in the menu selection’

Menu choice was associated with identity, which could be a barrier for those defining themselves as carnivores, or as facilitators for those privileging meat of quality, to the choice of the vegetarian menu. Dividing the issue into a binary problem — vegetarian or meat-eater — was seen as polarizing and unhelpful to the environment. The threat to identity was mainly a barrier to choosing the vegetarian menu in the cafeteria, and participants recommended approaching the topic with more nuances to encourage their choice of the vegetarian menu.

Participants defined themselves as flexitarians or carnivores, and ‘not vegetarians’: ‘*Again, just because you eat a vegetarian meal doesn’t mean you’re a vegetarian*’ (P6). For some participants, eating meat was part of their identity, and they liked meat, even if they were aware that they were eating too much of it: ‘*So for me it’s usually because I love meat and I eat a lot of meat, way too much meat*’ (P11). Others were low meat eaters, but high-quality meat eaters, which was a facilitator for the choice of the vegetarian menu: ‘*The only meat I buy is organic meat and I’m almost sure that the meat offered here is not organic and that’s what makes me less eager and go more to the vegetarian menu*’ (P8). Eating a vegetarian meal could be criticized by colleagues or family, and the criticism was uncomfortable: ‘*Oh no,* [talking about a situation in a restaurant where the most appealing menu was the vegan one], *this is unbelievable, I’m not going to take the vegan dish, am I?* (laughs*). I told my wife,* “*if I take the vegan dish, you won’t say it, eh*” (laughs)’ (P10).

The division between carnivores and vegetarians was feared. The issue was seen as emotional, with the risk of polarizing people’s behaviour: ‘*Yes, I think there is a rupture that is happening more and more. The goal is not to make people angry, but it is to make them want to* [change their habits]*. That’s the main word* [want to]*, and then I’d rather have 100% of the population eating vegetarian every other day than to have 50% of the population eating vegan and the other half stuffing themselves with meat*’ (P10).

Freedom of choice was paramount, and the idea of prompting messages suggesting a good way to eat was not well received: ‘*Personally, I am not too convinced by this kind of action* [about prompting messages]. *Especially if it becomes massive within a school, for example, I think it can very quickly lead to polarization, that’s clear. It can very quickly actually give the feeling that we are trying to impose a way of doing things. And I think it would be more intelligent, because we are in a school, to do it through the courses that are given to the students*’ (P7). Participants were aware of the need to reduce meat consumption: *‘ But here it is, the main idea is really, the awareness of saying I eat too much meat so if I have the opportunity to be able to eat vegetarian I try’* (P11), but attractiveness was seen as a superior facilitator of the choice of the vegetarian menu than restricting the choice by any measure: ‘*Mmmm no, we are already in a setting where we are told what to do all the time and if the only time when we are on a break and we can really choose, we can choose within the limits of the choices available, but if even there we are told what to do, I think it is useless, in fact, if precisely it’s not attractive*’ (P3). Time was seen as an important factor to consider in changing the behaviour of cafeteria customers: ‘*Because little by little they’re actually gonna try*’ (P9).

## Discussion

In this study, we were interested in the barriers and facilitators of consuming vegetarian menus in a university cafeteria. The quantitative results showed that the main barriers were the lack of vegetarian options, that the vegetarian menu did not taste good, and that it was not sufficiently filled. A large number of facilitators emerged from the questionnaire related to the health and environmental benefits of the vegetarian menu and the reduced price of the vegetarian menu for students. FG analysis revealed similar themes: at the motivational level, participants said that they chose their menu spontaneously and that the vegetarian plate was less attractive. Even for participants who wanted to do the right thing for their health or for the environment, which were facilitators also mentioned in the survey, choosing the vegetarian menu was not always easy because of its lack of attractiveness. In terms of opportunity, the lack of options and reduced prices emerged as barriers and facilitators, as in the survey. Environmental sensitivity, which emerged as a facilitator in the survey, turned out to be a more complex factor in FG, with some questioning whether eating animal protein was the right fight for the environment. Misperceptions or a lack of knowledge about what food is healthy or sustainable emerged from the FG as well as from the survey comments. Finally, participants expressed concern that a vegetarian menu information campaign would fuel the divide between vegetarians and meat eaters and expressed the need for time and choice, confirming some of the comments made in the survey.

According to the results of the first Swiss National Nutrition Survey, menuCH, 4.9% of respondents said they habitually eat a vegetarian diet, with more women than men, and more than 7% of people aged 18–34.^(27)^ In our study, 8% of the respondents followed a vegetarian diet, which can be attributed to the younger average age of our participants. This age-related trend has been observed in other studies^(27,28)^ and may be due to social influences, or environmental concerns among younger individuals. Therefore, our findings may not fully generalize to older populations with different dietary habits and motivations. Furthermore, as this is a convenience sample, it is possible that people who are more aware of environmental and health issues completed the survey.

Taste factors were also an important barrier to the acceptance of a plant-based diet in a school in Sweden for both adolescent students and teachers.^(29)^ Nevertheless, for Danish adults, the tastelessness of a plant-based diet was perceived as a barrier only for high-meat consumers, while the good taste of this type of dish was considered as a facilitator for low consumers of animal products,^(20)^ showing how pre-existing meat attachment influences responses. This phenomenon was also observed in our study, as the good taste of the vegetarian menu was considered a facilitator by women and the lack of flavour as a barrier by men, who are major consumers of meat.^(6)^ These findings suggest that targeted taste enhancement strategies may be essential to increase the attractiveness of vegetarian menus, particularly among demographic groups that are traditionally less inclined towards plant-based diets.

The lack of availability of plant-based options when eating out,^(30)^ or the difficulty to finding them in restaurants/cafes etc. or even in the supermarkets,^(20)^ was also mentioned as an obstacle in other studies. These barriers are consistent with the lack of vegetarian options cited by the participants in our study. Nudging interventions in the food environment, such as increasing the visibility and variety of vegetarian foods, have a positive effect on reducing meat consumption.^(31)^ It should be noted that the cafeteria at the University of Geneva, where our research was conducted, had fewer options at the time of the study due to the COVID-19 pandemic. In any case, even if there were more options (salad buffet and more than one vegetarian menu), the daily flow of customers was very variable, so increasing the number of vegetarian options would be difficult to manage, with the risk of increasing food waste. It would be interesting to investigate whether improving the attractiveness of the available vegetarian menu, coupled with a change in the appeal (e.g. ‘Chef’s suggestion’, ‘World cuisine’, and not using the word ‘vegetarian’), could be able to improve acceptance and attractiveness, regardless of the number of options available.

As a facilitator, a plant-based diet was considered to have health-related benefits for customers in Australia^(30)^ and this was also an argument for choosing a vegetarian menu for the respondents in our study. Nonetheless, although the participants were aware of the benefits of a plant-based diet, the FG responses highlighted the importance of the attractiveness of the plate and the importance of habits and customs in the final menu choice.

Similar to our results, the perception that the vegetarian menu was not sufficiently filled was identified as a relevant barrier to choosing sustainable foods also among university students in Finland^(15)^ and adults in Denmark.^(20)^ However, these studies did not report the average protein intake of participants. In any case, when satiety was compared between plant-based and animal-based meals, vegetarian and vegan meal choices did not explain differences in post-meal hunger, protein content marginally mediated post-meal satiety, while taste ratings had a strong effect on satiety and mood in general.^(32)^ In our study, the vegetarian menu was certified by a label guaranteeing varied and balanced meals (carbohydrates, proteins, and vegetables).^(33)^ In addition, registered dieticians analysed vegetarian menus over 8 weeks and confirmed that they were well-balanced. Customers also had the option of an extra portion if they were still hungry after finishing their vegetarian menus. Therefore, we can assume that a lack of satiety is a representation, particularly in men.

Moreover, Mäkiniemi *et al.* showed discrepancies between barriers perceived to climate-friendly food choices and barriers associated with final food choices.^(15)^ Participants may have based their responses to the barriers on their pre-existing and possibly inaccurate understanding of sustainable food choices, but they make habitual choices without reflection and offer post-hoc justifications for their choices.^(15)^ In our study, even though 82% of those surveyed intended to follow a sustainable diet, the final choice of a vegetarian menu seemed to be influenced by other factors (e.g. appearance, taste, price). Environmental awareness was more of a barrier than a facilitator to vegetarian menu choice in the FG, and some people compared the impact of meat consumption with the origin of grains or vegetables (local or foreign) used as ingredients in the vegetarian menu. Although disbelief in the effects of climate on food choices was not explicitly mentioned, other causes of global warming were evoked (e.g. plane travel), implying that other factors may affect the environment and that eating meat is not so serious.

Among the five themes identified in the qualitative analyses conducted by Eustachio Colombo *et al.*,^(29)^ some barriers are consistent with our analyses and may limit the choice of the low environmental impact menu, such as the role of sensory factors and habitual eating patterns in the acceptance of plant-based foods, the need to implement changes gradually, and the fact of being aware of the importance of sustainability, but the difficulty of integrating them into their dietary choices. Interestingly, kitchen staff expressed that they did not have training, financial resources, or adequate equipment to produce sustainable, good-quality meals.^(31)^ The kitchen team was not part of our study population, but it received regular training, including cooking vegetarian meals. It would have been interesting to ask them if they shared this opinion or what the barriers and facilitators were specific to the cafeteria in which they worked, especially considering that the vegetarian menu had to be sold to the students at half the normal price.

Concerning the difference between the barriers and facilitators mentioned by gender, in our study, women were more likely to report that there were no barriers to choosing a vegetarian menu, whereas men reported that nothing made it easier to choose a vegetarian option. Similarly, being a woman was positively associated and being a man was negatively associated with climate-friendly food choices among university students in Finland.^(15)^ Moreover, in agreement with our results, Lea *et al.* found that in Australia, more women than men believed plant-based diets could help the environment and animal welfare and provide a tastier diet, and more men than women cited as a barrier that a plant-based diet would not be tasty enough.^(30)^ Therefore, consideration of these differences is essential for the development of strategies to promote vegetarian menu sales.

Considering the differences between students and employees, the economic benefits and the link with the prevention of certain diseases were facilitators to choose the vegetarian menu respectively. Students were also more worried about the risk of losing muscle mass when choosing a plant-based menu. In Australia, age differences were more important than sex differences when the perceived barriers and benefits of adopting a vegetarian diet were investigated.^(19)^ In the university cafeteria in Geneva, a label guaranteed the presence of protein sources on the vegetarian menu: eggs, legumes, tofu, dairy products, soy-based drinks and dairy products, soy drinks, and yogurt, among others. Education activities could be a good strategy to address misperceptions or lack of knowledge about the composition of a balanced vegetarian menu and also to reinforce the knowledge of how it benefits health and the environment.

The barriers and facilitators to eating the vegetarian cafeteria menu appeared to differ between omnivores, who identified more barriers, and flexitarians and vegetarians/vegans, who identified more facilitators. Our findings are consistent with Mäkiniemi’s *et al.*^(15)^ study, which showed that vegetarians perceived barriers as less relevant than other participants. This highlights the need for targeted interventions to promote vegetarian options to groups that are more resistant to change, such as omnivores and men.

The limitations of this study include: (a) the cafeteria was still undergoing some post-COVID-19 measures at the time of the study, resulting in limited options like the removal of the salad bar; (b) the survey and interview guide for the FG were designed by our research team, which may affect the accuracy and consistency of the data collected; (c) the survey did not include a pre-submission completeness check or a step to review and amend responses before submitting; (d) the default settings of LimeSurvey® were used, including session cookies for user management without persistent unique identifiers and no IP address verification or restriction. While this decision could lead to potential duplicate submissions from the same users, data protection and privacy standards have been guaranteed; (e) the vegetarian menu was sold at a preferential price to students, which was a major facilitator for this group to purchase the vegetarian option; (f) despite three registrations for the first FG, only one participant attended. Thus, the session was conducted as an interview using the same guide, and the participant’s contributions were included in the analysis; (g) the target group of 20- to 25-year-olds was under-represented in the focus groups, with only two participants; and (h) the development of the study was constrained by time, as other interventions related to sustainable food were to be implemented in the cafeteria. Despite these limitations, the present study was conducted to explore consumers’ opinions on the barriers and facilitators of vegetarian menu choices. For this purpose, consumer opinion was not only collected quantitatively but also complemented by qualitative information gathered in the discussion groups. Additionally, there is no validated tool for assessing barriers and facilitators to the choice of vegetarian meals in cafeteria. Therefore, the survey and the interview guide were constructed based on a theoretical model, adapted it to the specific context and incorporated findings from available literature previously used to investigate similar issues. They were also tested and adjusted before use. The researchers conducting the study were independent and not affiliated with either the university or the cafeteria. Although the number of FG participants was limited, all voices were represented (students, teachers, research teams, and administrative/technical staff). The longer duration and face-to-face nature of the FG sessions, which required the sacrifice of the lunch break, may have made them less attractive to the younger students who mainly participated in the online survey. Finally, there were no other interventions in the cafeteria at the time of the study.

## Conclusion

Consumers need to change current food consumption patterns to help reduce the environmental impact in general and food-related GHG emissions in particular. Analysis of the barriers and facilitators that influence the choice of a vegetarian menu in this university cafeteria represents an important step in designing appropriate and informed strategies that are more likely to work. In our study, the environment was not a primary motivator in the choice of vegetarian menu, but the attractiveness of the menu was crucial.

Several strategies related to our findings have been used to influence meat consumption behaviour and/or intentions to reduce meat consumption. Improving the attractiveness of the vegetarian menu and providing knowledge about well-balanced vegetarian dishes, as well as the relationship between meat consumption, human and environmental health, and animal welfare, could make it easier to choose a vegetarian option. Informing consumers about the label that guarantees the balance of the vegetarian menu offered in the cafeteria and giving consumers time to make changes to their diet are important aspects to consider. The price of a vegetarian menu should be based on the quality of the ingredients and their attractiveness. Finally, strategies must pay particular attention to men and omnivores as they were the most resistant to choosing a vegetarian menu.

## Supporting information

Bertoni Maluf et al. supplementary material Bertoni Maluf et al. supplementary material

## Footnotes

1 The Nutri-score is a label that helps compare similar products. Products are ranked on a scale of five colours associated with letters from A to E (F does not exist), with letter A being the best nutritional composition, written in green, to letter E being the worst nutritional composition, written in red.

2 GRTA stands for Genève Région – Terre Avenir (Geneva Region – Land of the Future) and is a quality label based on four concepts: quality, proximity, traceability and equity.

## Abbreviations

**CHF:** Swiss Franc; **COM-B:** Capability, Opportunity, and Motivation Behaviour; **FG:** focus groups; **GHG:** greenhouse gas emissions; **GMOs:** genetically modified organisms; **GRTA:** Genève Région; Terre Avenir (Geneva Region; Land of the Future); **TDF:** Theoretical Domains Framework.

## Supplementary material

The supplementary material for this article can be found at https://doi.org/10.1017/jns.2024.69
