# Supplementary material for: Barriers and facilitators influencing the choice of a vegetarian menu in a university cafeteria
Source: J Nutr Sci. 2024 Nov 18;13:e71. doi: 10.1017/jns.2024.69 (PMC11588414; doi:10.1017/jns.2024.69)
Supplement: Bertoni Maluf et al. supplementary material [file S2048679024000697sup001.docx]

Supplementary Materials

**S1: Survey announcement sent by email to all students and staff members of the university:**

**Object of the email:** Do you often visit your university cafeteria? This message is for you!

**Main text of the email:**

Hello everyone,

We are a team from the Department of Nutrition and Dietetics at the Geneva School of Health Sciences.

Next autumn (2022), we plan to conduct an intervention in the cafeteria with the aim of promoting a more environmentally sustainable dietary approach. In order for the intervention to be relevant and tailored, we kindly ask you to complete this questionnaire to understand your perspectives on a more sustainable diet.

The estimated time to complete this questionnaire is 10 minutes. The more of you who complete it, the better we can tailor our messages.

If you have any questions, please feel free to contact Valeria Bertoni Maluf via email at valeria-andrea.bertoni-maluf@hesge.ch.

We appreciate the time you take and thank you in advance for your participation.

**S2: Online Survey regarding vegetarian menu in a university cafeteria in Geneva**

**Part A: Some questions about you** (Gender, age, and profession)

A1. What is your gender?

Female

Male

Other

A2. What is your age?

A3. You are:

Student

Teaching staff

Administrative and technical staff

**Part B: Types of diets** (e.g. omnivore, vegetarian, flexitarian)

B1. Please describe your type of diet as accurately as possible:

Omnivore (I eat everything)

Flexitarian (I consciously limit my meat consumption)

Vegetarian (I consume eggs and dairy products)

Vegan (I do not consume any animal products)

B2. Do you intend to adopt a diet that has a lower impact on the environment?

Yes

Rather yes

Rather no

No

B3. Is adopting a diet with a lower environmental impact aligned with your values?

Yes

Rather yes

Rather no

No

B4. Are you aware of which dietary choices are more environmentally friendly? The term "meat" refers to: red meat (beef, pork, lamb, veal, ...), poultry (chicken, turkey, duck, ...), and processed meats (ham, sausages, salami, ...).

Eating a diet with less meat

Consuming a diet with more plant-based proteins

Eating locally sourced foods

Eating seasonally

I pay close attention to food production (I look for labels)

I believe that at my scale, I don't have the ability to influence climate change

I don't know how it should be done

Other (free comments)

**Part C: Recommendations Regarding Meat and Fish Consumption**

C1. The Commission for Healthy and Sustainable Diets from the British scientific journal The EAT-Lancet recommends:

| **Type of meat or fish** | **Quantity** |
| --- | --- |
| Beef and lamb | A maximum of 50 grams per week |
| Pork | A maximum of 50 grams per week |
| Chicken and other poultry | A maximum of 200 grams per week |
| Fish | A maximum of 200 grams per week |

Please select the option that best describes your response:

I am not aware of these recommendations.

I find these recommendations surprising.

I am aware of the Lancet's recommendations, and I already follow them.

I am aware of the Lancet's recommendations, but I do not follow them.

I was not aware of the Lancet's recommendations, and I feel unable to follow them.

I find it difficult to visualize what these recommendations mean in practice.

Other (free comments)

C2. Considering the ETA-Lancet's recommendations, would you be willing to reduce your consumption of meat and fish?

I do not eat meat or fish.

I already pay very close attention to my consumption.

I would like to reduce my consumption, but I find it difficult.

I do not wish to reduce my consumption; I do other things for the planet.

I do not wish to reduce my consumption; it's too restrictive.

I do not wish to reduce my consumption; I derive too much pleasure from eating it.

Other (free comments)

**Part D: Barriers and Facilitators (**Barriers and facilitators to consuming the vegetarian menu at the cafeteria)

D1. What would be barriers for you to consume the vegetarian menu at the cafeteria? (multiple choices possible)

I enjoy eating meat or fish at the cafeteria.

Eating meat or fish at the cafeteria is financially accessible, so I take advantage of it.

I believe that humans are meant to eat meat and fish; it has been crucial for their evolution.

My peers eat meat/fish; everyone does it.

It's not that important for the planet.

Not eating meat/fish would make me worried about my health (e.g., deficiencies in iron, vitamin B12).

There are not enough proteins in a vegetarian diet.

I'm afraid of what others will think of me if I choose not to eat meat/fish.

I will lose muscle mass.

The vegetarian menu is too expensive considering the energy it provides.

The vegetarian menu tastes worse than dishes with meat/fish.

Only people who are concerned about their weight who don't eat meat.

Everyone eats meat/fish, why should I make an effort for the planet?

It's too hard to be the only one not ordering a burger when everyone else does.

I don't want to change my habits.

There are no barriers for me.

Other (free comments)

D2. Below are additional obstacles to choosing a vegetarian menu that may arise when you eat at the cafeteria. Check what may apply to you:

I need more information about the vegetarian diet.

The vegetarian options are too limited at the cafeteria.

I don't know what to substitute for meat/fish when I build my plate at the buffet.

I have so much on my mind when I arrive at the cafeteria that I forget my resolutions to eat more sustainably.

I lack the willpower to stop eating meat/fish.

I would need guidelines or mnemonic tricks to help me make choices (e.g., acronyms).

The vegetarian menu is not filling enough; I'll get hungry too quickly (lack of strength and energy).

I have so much on my mind when I arrive at the cafeteria that I won't add thinking about the planet.

When I see what others have chosen as their meals, I get influenced despite my resolutions to eat more sustainably.

The options for a climate-friendly diet are not well indicated; it's confusing!

I am convinced that no matter what I eat, the planet is headed for disaster.

I'm tired of being told what I should or shouldn't do; if I want meat, I'll eat it.

Eating a vegetarian menu is simply not a habit.

There are no obstacles for me.

Other (free comments)

D3. What are the benefits of eating the vegetarian menu at the cafeteria for you? (multiple choices possible)

The vegetarian menu allows me to eat more fruits and vegetables.

The quality of fats is better in the vegetarian menu.

Eating the vegetarian menu helps me control my weight.

It helps protect the rights and well-being of animals.

The vegetarian menu helps prevent certain diseases (heart disease, cancer).

The vegetarian menu promotes better health by reducing my consumption of chemicals, steroids, and antibiotics that are present in meat.

The vegetarian menu has a lower negative impact on the environment.

Eating the vegetarian menu reduces my risk of food poisoning.

Eating the vegetarian menu helps me be less aggressive or irritable.

There are no benefits for me.

Other (free comments)

D4. Among the options below, what could facilitate your choice of the vegetarian menu at the cafeteria? (multiple choices possible)

Since the vegetarian plate is often priced at 5 CHF, I choose it more regularly.

I easily go for the vegetarian buffet (or choose a vegetarian menu) because the wait is shorter at the cafeteria.

The vegetarian menu allows me to spend less money.

Eating the vegetarian menu helps me stay fit and full of energy.

The vegetarian menu increases the efficiency of food production and therefore ensures food security for the entire planet (ensuring that everyone has enough to eat).

It makes me feel happy/satisfied with myself to do something for the planet (environment or animal welfare).

The vegetarian menu results in a more flavorful diet.

Eating the vegetarian menu allows me to adhere to my religious beliefs.

The vegetarian menu helps the feminist cause (supporting the oppressed).

There is nothing that facilitates this choice for me.

Other (free comments)

D5. What could help you choose a more vegetarian diet at the cafeteria? (Open question)

**Part E: Participation in Focus Groups**

E1. Following the receipt of this questionnaire, we will be organizing three focus groups with students, teachers and administrative/technical staff. Two sets of discussion groups will take place, one in May 2022 and another in January 2023.

If you are willing to participate, please provide us with your email address, and we will contact you.

Thank you very much for your participation!

**S3: Interview guide of focus groups in order to complete the survey responses, identify what needed to change to promote the choice of the vegetarian menu in the cafeteria, and select the most appropriate intervention**

Presentation and roles.

Acknowledgments and presentation of survey results.

We would like to hear your views on what information would be useful to you when choosing a menu in the cafeteria.

All ideas are welcome in this discussion group, so please listen respectfully to what everyone has to say and allow everyone to express themselves.

1. Firstly, do you recognise yourself in these general findings or do you have anything to add?
2. When you eat in the cafeteria, do you choose or try to choose a diet that has less impact on the environment? **(memory, decision process)**
   1. Does it sometimes involve choosing the vegetarian menu?
   2. If not why?
   3. What else do you do?
3. What is your main motivation for eating a vegetarian menu when you do?
   1. Does the price matter? (If not mentioned for the students)
4. Can eating the vegetarian menu generate an emotional response in you? For example, does it make you feel satisfied that you are helping the environment, or does it make you worry that you are not eating a balanced diet? **(emotion)**
5. How easy or difficult is it for you to choose a more sustainable menu at the cafeteria? **(context, capabilities)**
6. Are the vegetarian options clearly marked? **(context/capabilities)**
7. Would it motivate you if it was clearly labelled?
8. What would you suggest as a name for the vegetarian menu? For example: sustainable plate, etc.)
9. What kind of information would help you:
   1. About what?
   2. Would it be helpful to provide information on origin (local), source or amount of protein?
   3. What kind of information is currently available in the cafeteria?
10. Do you know what GRTA means? (GRTA label, Genève Région terre Avenir)
11. What is the best way to present information? (labels on dishes, menu sheets, etc.)
12. What is missing from the current information? What do you like or dislike?
13. If new information is developed, how can it help you make informed cafeteria choices?
14. What information is currently missing? How should it be displayed? (information sheets displayed, acronyms/logos, flyers on tables, GRTA stand, ...)
15. Is there anything else that could help you get more information?
16. As someone who knows the cafeteria's clientele, what do you think would be useful and acceptable to users to promote the vegetarian menu, while still allowing freedom of choice?
17. Do you think, for example, that more general messages of encouragement could achieve their aim, or could they increase opposition? (For example, eating vegetarian occasionally makes you happy to do something for the environment, ...)
18. Do you have anything else to add to this topic?

Thank you very much for your participation.

Provide 20 CHF vouchers at the cafeteria.

Mention the FG for next year: In a year, we would like to hold discussion groups again to see if any changes have occurred. Would you be available and open to the idea of us contacting you again?

**Supplementary Table 1**. Barriers to choosing the vegetarian menu in the cafeteria, by position (total n=304, n=195 students and n=109 employees)

| Barriers/Obstacles^a^ | All^b^ n=304 (100%) | Students n=195 (100%) | Employees n=109 (100%) | p value^b^ |
| --- | --- | --- | --- | --- |
| No barriers | 124 (40.8) | 74 (37.9) | 50 (45.9) | 0.183 |
| Too few vegetarian options | 129 (42.4) | 82 (42.1) | 47 (43.1) | 0.904 |
| Tastes less good | 92 (30.3) | 62 (31.8) | 30 (27.5) | 0.515 |
| Insufficiently satiating | 72 (23.7) | 50 (25.6) | 22 (20.2) | 0.326 |
| Enjoy eating meat or fish in the cafeteria | 59 (19.4) | 41 (21.0) | 18 (16.5) | 0.368 |
| High price in relation to energy provided | 47 (15.5) | 35 (17.9) | 12 (11.0) | 0.136 |
| Worry about deficiencies (iron, vit. B12) | 44 (14.5) | 32 (16.4) | 12 (11.0) | 0.236 |
| Worry about not getting enough protein | 43 (14.1) | 34 (17.4) | 9 (8.3) | 0.038* |
| Take advantage of eating meat or fish in the cafeteria because it is affordable | 41 (13.5) | 35 (19.7) | 6 (5.5) | 0.003* |
| Don't have the habit | 36 (11.8) | 19 (9.7) | 17 (15.6) | 0.141 |
| Tired of being told what to do and what not to do | 35 (11.5) | 19 (9.7) | 16 (14.7) | 0.260 |
| Think that human beings were meant to eat meat and fish, to evolve | 34 (11.2) | 23 (11.8) | 11 (10.1) | 0.708 |
| Whatever your diet, the planet will not be saved | 34 (11.2) | 19 (9.7) | 15 (13.8) | 0.343 |
| Lack of willpower to stop meat/fish consumption | 28 (9.2) | 21 (10.8) | 7 (6.4) | 0.301 |
| Lack of knowledge about the substitution of meat/fish at the buffet | 23 (7.6) | 17 (8.7) | 6 (5.5) | 0.371 |
| Fear of losing muscle | 20 (6.6) | 19 (9.7) | 1 (0.9) | 0.003* |
| Having so many things on your mind that by the time you get to the cafeteria, you forget your resolutions to eat more sustainably | 19 (6.3) | 11 (5.6) | 8 (7.3) | 0.624 |
| Other answers | < 5% |  |  |  |

^a^ More than one answer was possible. ^b^ Fisher’s exact test. *P<0.05.

**Table 2**. Facilitators to choosing the vegetarian menu in the cafeteria, by position (total n=304, n=195 students and n=109 employees)

| Facilitators^a^ | All^b^ n=304 (100%) | Students n=195 (100%) | Employees n=109 (100%) | p value^b^ |
| --- | --- | --- | --- | --- |
| Eat more fruit and vegetables with the vegetarian menu | 170 (55.9) | 106 (54.4) | 64 (58.7) | 0.473 |
| Less environmental impact | 154 (50.7) | 106 (54.4) | 48 (44.0) | 0.095 |
| As the vegetarian menu is often priced at CHF 5, I take it more regularly | 127 (41.8) | 110 (56.4) | 17 (15.6) | <0.001* |
| Allows you to spend less money | 117 (38.5) | 91 (46.7) | 26 (23.9) | <0.001* |
| Helps to improve health through the reduction of negative products in meat (chemicals, steroids and antibiotics) | 88 (28.9) | 50 (25.6) | 38 (34.9) | 0.113 |
| Contributes to animal rights and animal welfare | 87 (28.6) | 57 (29.2) | 30 (27.5) | 0.792 |
| The satisfaction of doing something for the planet (the environment or the welfare of animals) | 80 (26.3) | 54 (27.7) | 26 (23.9) | 0.499 |
| The quality of the fat is better | 73 (24.0) | 45 (23.1) | 28 (25.7) | 0.675 |
| Helps to prevent certain types of disease | 65 (21.4) | 34 (17.4) | 31 (28.4) | 0.029* |
| Improvement in the efficiency of food production and hence food security | 55 (18.1) | 33 (16.9) | 22 (20.2) | 0.535 |
| Nothing makes that choice easier | 54 (17.8) | 25 (12.8) | 29 (26.6) | 0.004* |
| Allows you to control your weight | 44 (14.5) | 26 (13.3) | 18 (16.5) | 0.498 |
| Helps you to stay fit and full of energy | 40 (13.2) | 23 (11.8) | 17 (15.6) | 0.378 |
| No profit | 28 (9.2) | 15 (7.7) | 13 (11.9) | 0.223 |
| Allows you to have a tastier diet | 27 (8.9) | 16 (8.2) | 11 (10.1) | 0.675 |
| Choose a vegetarian option as the wait in the cafeteria is shorter | 20 (6.6) | 8 (4.1) | 12 (11.0) | 0.028* |
| Other answers | < 5% |  |  |  |

CHF, Swiss Franc. ^a^ More than one answer was possible. ^b^ Fisher’s exact test. * P<0.05.
